# Ameliorative Effects of Infantile Feire Kechuan Oral Solution on Mycoplasma Pneumoniae Pneumonia in Infant Mouse and Rat Models

**DOI:** 10.1155/2018/8139040

**Published:** 2018-09-17

**Authors:** Shanshan Guo, Lei Bao, Tiange Qu, Xin Mao, Yingjie Gao, Yingli Xu, Xiaolan Cui

**Affiliations:** ^1^Institute of Chinese Materia Medica, China Academy of Chinese Medical Sciences, No. 4 Yinghua East Road, Chaoyang District, Beijing 100029, China; ^2^Beijing University of Chinese Medicine, No. 11, Bei San Huan Dong Lu, Chaoyang District, Beijing 100029, China

## Abstract

*Mycoplasma pneumoniae* (MP) infection is a major pathogen of community-acquired pneumonia (CAP) in children worldwide. Infantile Feire Kechuan Oral Solution (IFKOS) has been used for the treatment of MP pneumonia clinically in China for many years. The present study was designed to investigate the therapeutic effect of IFKOS on MP pneumonia and explore the potential mechanism of the actions. The infant BALB/c mouse and Wistar rat models of MP infection were successfully established to confirm the therapeutic effects of IFKOS, followed by assays for related cytokines and investigations of the IgM response involved. The results showed that IFKOS exhibited an inhibitory effect on pulmonary index (PI) and effectively reduced the degree of lesions in the lungs. The lethal rate of mice was significantly decreased while survival time of mice was dramatically increased by IFKOS treatment in comparison to infection control, respectively. IFKOS treatment (40, 20, and 10ml/kg) significantly decreased the level of MP-IgM in a dose-dependent manner, whereas IFKOS showed no obvious inhibitory effect on the increase of relative expression of MP-DNA. In addition, the elevated IL-2 and TNF-*α* levels were significantly reduced and the decreased IL-6 level was significantly enhanced by IFKOS treatment. Our study demonstrates that IFKOS has inhibitory effect on MP infection in infant mouse and rat models of MP pneumonia and protective effect from lethal MP challenge in infant murine model. These anti-MP effects might be related to suppression of the IgM response and a reversal the imbalance of Th1/Th2 cytokines induced by MP infection.

## 1. Introduction 

*Mycoplasma pneumoniae* (MP) is one of the most common pathogens of community- acquired pneumonia (CAP) in young adults and children and is a significant worldwide cause of morbidity and mortality in both adult and pediatric populations [1]. MP accounts for 3–10% of pneumonia cases and 10-30% of all CAP cases [2, 3], especially in east China, and MP infection has been detected in approximately 50% of the CAP population [4]. Although MP pneumonia in most patients is generally mild or moderate, with only 3%-13% of infected persons developing pneumonia, in recent years increasing numbers of severe pneumonia in children from MP infection have been reported, including many fatal cases [5, 6]. MP not only causes upper and lower respiratory tract infections, but also leads to extrapulmonary complications which affect almost every organ [7].

It has been demonstrated that an excessive host immune response against pathogens may play a pivotal role in refractory* Mycoplasma pneumoniae *pneumonia (RMPP) in school-aged children, including overexpression of cytokines, such as interleukin-1ß (IL-1 ß), interleukin-6 (IL-6), interleukin-10 (IL-10), interferon gamma (IFN-*γ*), tumor necrosis factor alpha (TNF-*α*), and lactate dehydrogenase (LDH) [8–10]. The severity of* Mycoplasma pneumoniae *lung disease may be related to the degree to which the host immune response reacts to the infection, based on evidence from clinical research and from studies in vitro and in vivo [11]. It was found that IL-8, IL-10, and IL-18 appeared to modulate pulmonary lesions in MP pneumonia in children of a median age of 5 years [12]. In addition, the production of IL-2, IL-4, IL-10, and IFN-*γ* by splenic lymphocytes has been detected in significant high levels in a gnotobiotic mice model of* Mycoplasma pneumoniae* pneumonia, and IL-1ß and IL-6 were upregulated in a novel mouse model immunized with MP extracts plus alum [13, 14].

Macrolides have been recommended as the first-line therapy in adults, whereas azithromycin (AZM) as the first-line drug in for pediatric cases [15, 16]. However, macrolide-resistant MP pneumoniae (MRMP) pneumonia have emerged and became widespread in East Asia since 2000. In the last decade, macrolide resistance rates in MRMP pneumoniae among children have substantially increased worldwide [17, 18], especially in China and Japan, where it has reached up to 90% [19, 20]. Therefore, it is extremely urgent to develop new drugs as safe and effective alternative agents for the treatment of MP pneumonia.

Infantile Feire Kechuan Oral Solution (IFKOS) is a traditional Chinese medicine that is mainly used to treat respiratory tract infections such as influenza and pneumonia in children. IFKOS is composed of the following Chinese medicines,* Lonicerae japonicae Flos, Ephedrae Herba, Glycyrrhizae Radix et Rhizoma, Forsythiae Fructus, Scutellariae Radix, *and* Armeniacae Semen amarum*. Studies have reported that active constituents of IFKOS such as chlorogenic acid and forsythiaside may have pharmacological functions of wind dispelling, heat-clearing, detoxifying, and pain relieving [21, 22]. In China, IFKOS has been listed as a proprietary Chinese medicine to combat pediatric respiratory infection. Many clinical studies have reported the curative effects of IFKOS against MP pneumonia, and the cure rate was up to 90.70% when infantile MP pneumonia was treated with IFKOS combined with azithromycin [23–25]. However, few studies have investigated the curative and underlying mechanisms of the actions involved in experimental models.

In the present study, the therapeutic effect and mechanisms of IFKOS on MP pneumonia were assessed using infancy BALB/c mouse and Wistar rat models. Quantitative real-time polymerase chain reaction and an enzyme-linked immunosorbent assay (ELISA) were used to evaluate the therapeutic effect of IFKOS on the expression levels of MP-DNA, MP-IgM, IL-2, IL-6, and TNF-*α* in response to MP pneumonia. In addition, pulmonary injury in mice and rats was detected by pathological examination under microscope and the mortality rate and survival time in a BALB/c mice model were observed.

## 2. Materials and Methods

### 2.1. Test Drugs

All investigations were performed with a single batch (No. 201610001) of IFKOS kindly provided by the manufacturer Kuihua Pharmaceutical (Heilongjiang, China). The doses of IFKOS were 40, 20, and 10ml/kg/day for infant mice and 20, 10, and 5ml/kg/day for infant rats. All the doses for mice and rats were equivalent to two, one, and half times the clinical dose of 6-year-old children (2ml/kg) according to the ratio of body surface area of human and mice or rats, respectively. Azithromycin (batch No. 063160821) was produced by Ouyi Pharma, China Shijiazhuang Pharmaceutical Group, served as a reference drug. The therapeutic dose of azithromycin is 5 mg/kg daily (15-25kg children), and the doses for mice and rats were 50 and 25mg/kg/day according to the ratio of body surface area of human and mice or rats, respectively.

### 2.2. Animals

Male and female BALB/c mice (13-15g) and Wistar rats (90-110g) were purchased from Vital River Laboratory Animal Technology Co., Ltd., in Beijing, China (Certificate No. SCXK 2016-0011) and maintained in an environmentally controlled breeding room (temperature: 20 ± 2°C, humidity: 50 ± 10%). The study was carried out in accordance with the rules of European Community Guidelines for care and use of animals. The study was approved by Ethics Committee at Institute of Chinese Materia Medica, China Academy of Chinese Medical Sciences.

### 2.3. MP Culture

MP, FH strain (ATCC15531, American Type Culture Collection, Rockville, MD, USA) was cultured in modified mycoplasma medium (Oxoid, UK) containing PPLO broth, horse serum, and 25 % yeast extract, to which was added penicillin G (200 U/ml), thallium acetate (0.025 %), glucose (1 %), and phenol red (0.002 %), pH 7.8. MP was cultured at 37°C under 5 % CO_2_ for 7 days. The concentration of MP was determined as 10^8^ color change units (CCU)/ml, according to the color-change-unit method [26].

### 2.4. Mouse Model of MP Pneumonia

BALB/c mice were randomly divided into six groups with 10 mice per group (half male and half female). The groups were assigned as normal control group, infection control group, and four treatment groups (three IFKOS treatment groups and one positive drug-azithromycin treatment group). All mice except those in the normal control group were repeatedly infected intranasally with 50*μ*L of the MP solution at a concentration of 10^8^CCU/ml for three times, once a day. In the treatment groups, the inoculated mice were successively given IFKOS orally at a dose of 40ml/kg, 20ml/kg, or 10ml/kg or treated with azithromycin at a dose of 50mg/kg daily for 3 days beginning from the day of infection. Each mouse of the control group was given an equal volume of distilled water. On day 4, blood of all the mice was collected and the serum was separated to determine the level of MP-IgM. Then the mice were sacrificed, and the body weight (BW) and lung weight (LW) were measured to calculate the pulmonary index (PI) and inhibitory rate of pulmonary indices (IRPI).PI=[LW(g)/BW(g)]×100,IRPI=[(LW of infection control group-LW of treatment group)/(LW of infection control group-LW of normal control group)]×100. The lung tissues were extracted and subjected to qRT-PCR detection and histological examination.

### 2.5. Rat Model of MP Pneumonia

Wistar rats were randomly divided into six groups with 10 rats per group (half male and half female) as described above. All rats except those in the normal control group were repeatedly infected intranasally with 150*μ*L of the MP solution at a concentration of 10^8^CCU/ml for four times, once a day. In the treatment groups, the inoculated rats were successively given IFOKS orally at a dose of 20ml/kg, 10ml/kg, or 5ml/kg, or treated with azithromycin at a dose of 25mg/kg daily for 4 days beginning from the day of infection. Each rat of the control group was given an equal volume of distilled water. On day 5, all the rats were sacrificed, and the lung tissues were extracted and subjected to ELISA assay and histological examination.

### 2.6. Mouse Model of Death due To MP Infection

A total of 100 BALB/c mice were randomly distributed into five groups with 20 mice per group, including infection control group, azithromycin treatment group, and three IFKOS treatment groups. All the mice were repeatedly infected intranasally with 60*μ*L of the MP solution at a concentration of 10^8^CCU/ml for five times, once a day. In the treatment groups, the inoculated mice were successively given IFKOS orally at a dose of 40ml/kg, 20ml/kg, or 10ml/kg or treated with azithromycin at a dose of 50mg/kg daily for 5 days at the beginning of the infection day. Each mouse of the control group was given an equal volume of distilled water. The day after the last infection, the number of deaths in each group was recorded for 14 consecutive days.

### 2.7. Quantitative Real-Time PCR Detection of MP-DNA

DNA samples were extracted from the left lobes of all mice lungs using the DNeasy Blood & Tissue kit (Qiagen, Hilden, Germany) according to the manufacturer's protocol. The presence of MP*-*DNA was diagnosed using MP Real-Time PCR kit (Zhi-Jiang Co., Ltd, Shanghai, China) with the following PCR amplification system: DNA template (4*μ*L), MP PCR reaction mix (35*μ*L), and Taq enzyme (0.4*μ*L). In the real-time PCR, SYBR green was used as fluorescence reporter, specific forward primer 5′-CAGAAACACACACAGCAGCTATT-3′ and reverse primer 5′-CACGTTGATCCGCAAAGGAAGT-3′. The PCR reaction was carried out using a quantitative PCR instrument (Bio-Rad CFX96, USA) under the following conditions: 37°C for 2 min, 94°C for 2 min, followed by 40 cycles of 93°C for 15 s, and 60°C for 1 min.

### 2.8. ELISA Assay for MP-IgM, IL-2, IL-6, and TNF-*α*

The level of MP-IgM in mice serum was determined by an enzyme-linked immunosorbent assay(ELISA) kit (96T, Meilian Co., Ltd, Shanghai, China) according to the manufacturer's instructions, and the levels of IL-2, IL-6, and TNF-*α* in rat lung tissues were measured using ELISA kits (96T, Meilian Co., Ltd., Shanghai, China).

### 2.9. Histological Examination

The right lobes of the lungs in mice or rats were fixed in 10% paraformaldehyde, then trimmed, and embedded in paraffin and cut into slices (3 mm) for hematoxylin and eosin (H&E) staining. Morphometric analysis was performed using an optical microscope (Leica DMLB-HC, Germany) to evaluate the histopathological changes of the lungs.

The following pulmonary infection score system was applied for grading the pulmonary lesion:

“0”: pulmonary alveoli showing no fusion, no alveolar interstitial infiltration of inflammation cells, no inflammation cells around the bronchi, and no shedding in the lumen.
“1”: pulmonary alveoli showing no fusion, but with localized pulmonary interstitial infiltration of lymphocytes and macrophagocytes inflammation cells, limitation inflammation cells around the bronchi, and no shedding in the lumen.
“2”: pulmonary alveoli showing no obvious enlargement, mild pulmonary interstitial infiltration of lymphocytes and macrophagocytes inflammation cells, mild inflammation cells around the bronchi, and no shedding in the lumen.
“3”: obvious pulmonary interstitial infiltration of lymphocytes and macrophagocytes inflammation cells, pulmonary interval widened, significant inflammation cells around the bronchi, and no obvious shedding in the lumen.
“4”: a large area of diffuse pulmonary interstitial infiltration of lymphocytes and macrophagocytes inflammation cells, pulmonary interval widened, a large area of diffuse inflammation cells around the bronchi, and no obvious shedding in the lumen.

### 2.10. Statistical Analysis

The GraphPad Prism 6.0 software system was employed for statistical analysis.

All data are presented as mean±SEM (standard error of the mean). A *χ*^2^ test was used to statistically analyze the reduction in mortality, other results were analyzed by one-way analysis of variance (ANOVA), and significant differences were determined by the Bonferroni Test.* P* values≤0.05 were taken as statistically significant.

## 3. Results

### 3.1. IFKOS Inhibits MP-Induced High Pulmonary Index (PI) in Mice

As shown in Table 1, mice from the infection control group presented an increase in PI (1.56±0.32) compared to the normal control group (0.68±0.12). After 3-day treatment, there was an obvious decrease of PI in all three IFKOS groups compared with the infection control group. In addition, IFKOS exhibited significant PI inhibitory activity, and the inhibitory rate of pulmonary indices (IRPI) were 54.93%, 31.57%, and 37.78% at the doses of 40, 20, and 10ml/kg, respectively.

### 3.2. Anti-*Mycoplasma pneumoniae* Activity of IFKOS

**Figure 1:**
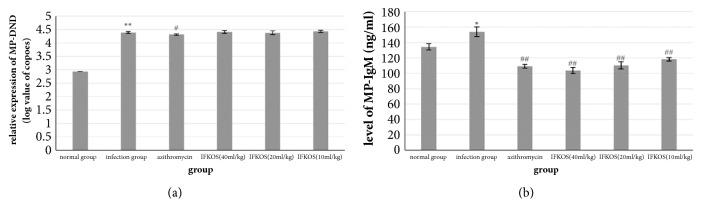
Anti-*Mycoplasma pneumoniae* activity of IFKOS in infancy BALB/c mice model. (a) The relative expression of MP-DNA was measured by qRT-PCR. Relative log values of MP copies were expressed as the mean ± SEM (n=6). ^*∗∗*^*p* < 0.01 compared to normal control group; ^#^*p* < 0.05 compared to infection control group. (b) The level of MP-IgM was measured by ELISA assay. Each value was represented as mean± SEM (n=10), ^*∗*^*p* < 0.05 compared to normal control group, ^##^*p* < 0.01 compared to infection control group.

In the current study, the anti-MP activity of IFKOS was measured using MP-DNA and MP-IgM in lungs or serum. To determine whether the replication of MP was inhibited by IFKOS directly in mice, we further examined the relative expression of MP-DNA. In addition, the level of MP-IgM in serum was detected to discover the effect of IFKOS on immune response induced by MP infection. As shown in Figures 1(a) and 1(b), the relative expression of MP-DNA increased dramatically in the infection control group, whereas IFKOS showed no obvious inhibitory effect on the increase of relative expression of MP-DNA, which demonstrates that MP replication in mice was not inhibited directly by IFKOS treatment. The level of MP-IgM in the infection group increased significantly compared to the normal control group. IFKOS treatment (40, 20, and 10ml/kg) significantly decreased the level of MP-IgM in a dose-dependent manner, which indicated that elevation of MP-specific IgM was significantly depressed by IFKOS treatment in a dose-dependent manner.

### 3.3. Inhibitory Effect of IFKOS on MP Pneumonia in Mice and Rats

**Figure 2:**
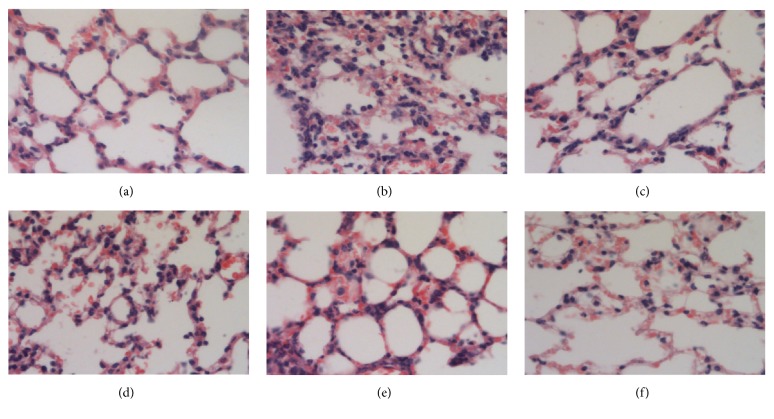
Pathological changes in lung tissues of BALB/c mice. After treatment with IFKOS for 3 days, the right lobes of the lungs in mice were fixed in 10% paraformaldehyde, embedded in paraffin, and cut into slices for H&E staining. Morphometric analysis was performed using an optical microscope. (a) Normal control group, (b) infected control group, (c) azithromycin treatment group, (d) IFKOS (40ml/kg) treatment group, (e) IFKOS (20ml/kg) treatment group, and (f) IFKOS (10ml/kg) treatment group.

After MP pneumonia mice were treated with IFKOS (40, 20, and 10ml/kg) for 3 days, the lung tissues were isolated for H&E staining. As shown in Figures 2(a)–2(f), in the normal control group, the lung tissues were wholly unaffected and there were no lesions in pulmonary alveolus and alveolar interstitium. In the infection group, a large area of diffuse pulmonary interstitial infiltration of lymphocytes and macrophagocytes inflammation cells was observed as well as widened pulmonary interval. In the IFKOS treatment group, the pulmonary interstitial infiltration of inflammation cells was decreased. As shown in Table 2, the score of lesions in the lungs was significantly reduced in mice treated with IFKOS at the dose of 10ml/kg.

**Figure 3:**
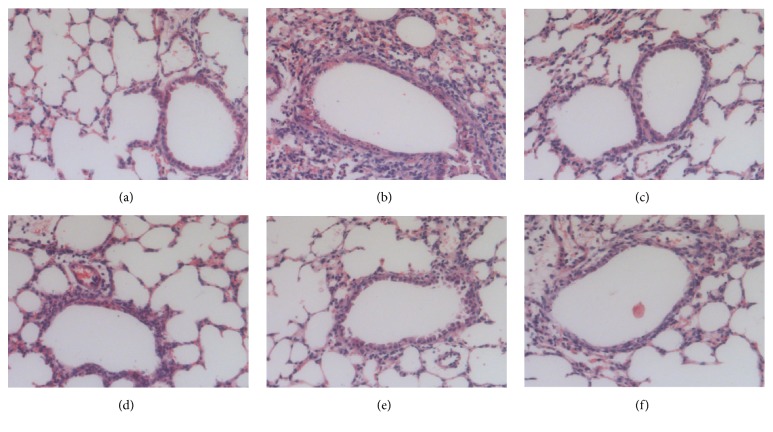
Pathological changes in bronchi of BALB/c mice. (a) Normal control group, (b) infected control group, (c) azithromycin treatment group, (d) IFKOS (40ml/kg) treatment group, (e) IFKOS (20ml/kg) treatment group, and (f) IFKOS (10ml/kg) treatment group.

As shown in Figures 3(a)–3(f), in the normal control group, there were no inflammation cells around the bronchi and no shedding in the lumen. In the infection group, significant inflammation cells were observed around the bronchi but without any obvious shedding in the lumen. However, as shown in Table 2, no significant effect of IFKOS treatment on bronchial lesion was seen.

After MP pneumonia rats were treated with IFKOS (20, 10, and 5ml/kg) for 4 days, histopathological examination was conducted to reveal significant pathological changes in lungs of rats. As shown in Figures 6(a)–6(f), in the normal control group, lung tissue retained its basic structure. In the infection group, pulmonary interstitial infiltration of lymphocytes and macrophagocytes inflammation cells was noted in alveolar interstitium and the pulmonary interval was widened. In the IFKOS treatment group, the pulmonary interstitial infiltration of inflammation cells was decreased. As shown in Table 2, the score of lesions in the lungs was significantly reduced when treated with IFKOS at the doses of 20 and 10ml/kg.

### 3.4. IFKOS Treatment Protects Mice from Lethal MP Challenge

**Figure 4:**
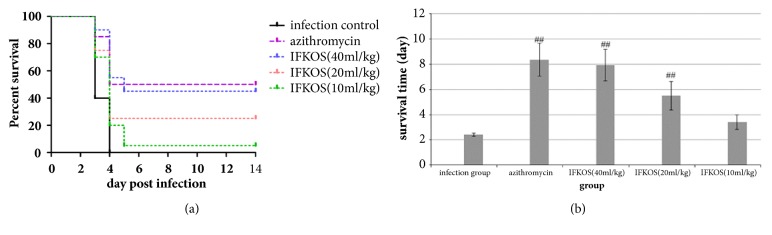
Protective effect of IFKOS on BALB/c mice from lethal MP challenge. The mice were repeatedly infected with 60*μ*L MP (10^8^CCU/ml ) for five times, once a day. After treatment with IFKOS for 5 days, the number of deaths in each group was recorded for 14 consecutive days. (n=20). (a) Survival rate of MP infected mice treated with IFKOS (40, 20, and 10ml/kg) in the 14 consecutive days. (b) IFKOS increased survival time (day) of mice in a dose-dependent manner. ^##^*p*<0.01 compared to infection control group.

To evaluate the protective efficacy of IFKOS against lethal MP challenge, BALB/c mice were subjected to sequential infection for five times. As shown in Figure 4(a), in the 14 days after the last infection, all the mice in the infection group died. The lethal rate was significantly decreased to 55% by IFKOS treatment at a dose of 40ml/kg compared to infection control. Furthermore, IFKOS treatment protected 9/20, 5/20, and 1/20 mice (45%, 25%, 5%) from death at the doses of 40, 20, and 10ml/kg, respectively. As shown in Figure 4(b), IFKOS treatment (40 and 20ml/kg) dramatically increased survival time of mice to 7.95 d and 5.50 d in comparison to the infection control, respectively.

### 3.5. Effect of IFKOS on Cytokines Secretion Induced by MP

**Figure 5:**
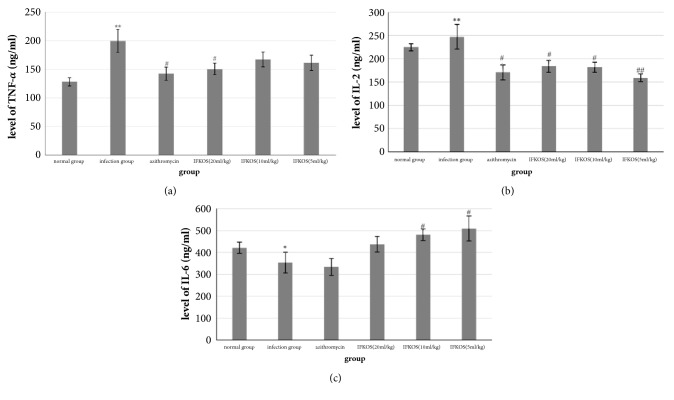
Effect of IFKOS on levels of cytokines in lung tissues of infancy rats infected by MP. Values were expressed as the mean ± SEM (n=10), ^*∗∗*^*p* < 0.01, ^*∗*^*p* < 0.05 compared to normal control group, ^##^*p* < 0.01, ^#^*p* < 0.05 compared to infection control group. (a) The level of TNF-*α*. (b) The level of IL-2. (c) The level of IL-6.

**Figure 6:**
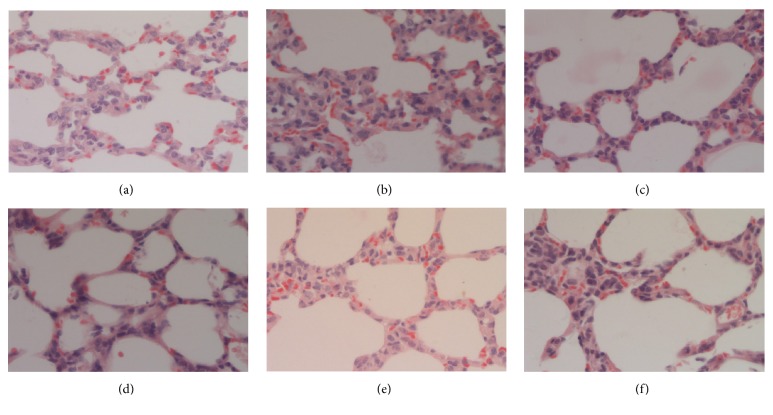
Pathological changes in lung tissues of Wistar rats. After treatment with IFKOS for 4 days, the right lobes of the lungs in rats were fixed in 10% paraformaldehyde, embedded in paraffin, and cut into slices for H&E staining. Morphometric analysis was performed using an optical microscope. (a) Normal control group, (b) infected control group, (c) azithromycin treatment group, (d) IFKOS (20ml/kg) treatment group, (e) IFKOS (10ml/kg) treatment group, and (f) IFKOS (5ml/kg) treatment group.

Many cytokines play a crucial role in inflammatory responses triggered by MP. To determine whether IFKOS exhibited anti-MP pneumonia activity by inhibiting cytokine secretion, IL-2, IL-6, and TNF-*α* in lung tissues were determined by ELISA. As shown in Figure 5, the levels of IL-2 and TNF-*α* were significantly increased while the level of IL-6 was obviously decreased in the infection group compared with the normal control group. However, IFKOS treatment (20,10, 5ml/kg) significantly decreased the level of IL-2 in a dose-dependent manner. The elevated TNF-*α* level was significantly decreased by IFKOS at the dose of 20ml/kg. In addition, a notable increase of IL-6 level was observed in the IFKOS (10, 5ml/kg) groups in comparison with the normal control group. These results demonstrate that cytokine secretion induced by MP infection was significantly reversed by IFKOS treatment.

## 4. Discussion

Although MP pneumonia is generally benign and self-limiting, several studies have indicated that MP can progress into severe life-threating diseases such as refractory MP pneumonia, acute respiratory distress syndrome, necrotizing pneumonitis, and fulminant pneumonia in school-aged children from 5 to 15 years [27, 28]. However, as overall macrolides have been extensively used in children, the occurrence of MRMP has led to more severe cases requiring hospitalization worldwide [29]. The main mechanism of resistance is mutations at the sites 2063, 2064, 2067, and 2617 in mutations in domain V of 23S rRNA [30]. MRMP pneumonia causes more severe clinical outcomes in children, including a longer duration of fever, cough, and hospital stay in addition to radiologic progression [15]. Thus, there is a high unmet medical need for MP pneumonia treatment for children, one which is effective for severe and fulminant disease.

Research on MP pneumonia usually focuses on detecting the physiological and biochemical characteristics of the patients and clinical treatment experience. Fewer studies have reported on the effects of traditional Chinese medicine on experimental animal models of MP infection. IFKOS has been used to treat MP pneumonia for many years in China, and clinical results have showed that this drug could effectively alleviate its symptoms and could also reduce the fever, cough, radiologic progression disappearance time, and the average hospitalization time in children [31, 32]. The present study was designed to demonstrate the anti-MP activity of IFKOS and to investigate the pharmacological mechanism of anti-MP pneumonia action.

In this study, we successfully established the infant mouse and rat models of MP infection according to reports in the literature [33, 34]. The therapeutic effects were confirmed in the animal models, followed by assays for related cytokines and investigations of the IgM response involved.

The results demonstrate that in the infant mouse model IFKOS exhibited an inhibitory effect on pulmonary index, indicating that IFKOS protected the lung from MP-induced injury. In the lung tissues of both infant mouse and rat models, a large amount of pulmonary interstitial infiltration of lymphocytes and macrophagocytes was observed as well as significant inflammation cells around the bronchi, which indicated the characteristic pathological feature of MP pneumonia [35]. IFKOS effectively reduced pulmonary interstitial infiltration of lymphocytes and macrophagocyte, and the degree of lesions in the lungs was significantly reduced in mouse and rat models. This study therefore provides direct evidence that IFKOS may represent a potential therapeutic agent for MP pneumonia.

Anti-*Mycoplasma pneumoniae*-specific immunoglobulin M (MP-IgM) is an indicator of early acute infection that can persist for several months in patients. MP-IgM typically appears within 1 week of the initial infection and approximately 2 weeks before MP-IgG, so that elevation of MP-IgM alone can often be interpreted as evidence of acute infection [36, 37]. We have detected the expression of specific MP-IgG in MP-mice model, but the level of MP-IgG in the infection group has no obvious change compared to the normal control group. IFKOS treatment (40, 20, and 10ml/kg) showed no significant effect on the level of MP-IgG. The presence of IgM is considered most significant in pediatric populations, and our mice model is in the early phase of MP infection, so the results of MP-IgG were not shown in the manuscript. Studies have demonstrated that the positive rate of IgM and IgA in* Mycoplasma pneumoniae *increased to 97.5 and 56.3%, respectively, at one week after infection. Compared with MP-IgA, MP-IgM has higher sensitivity in the diagnosis of mycoplasma-associated pneumonia in newborns, school-aged children, and adolescents [38, 39].* Mycoplasma pneumoniae* affects the immune responsiveness of the host is their propensity for mitogenic stimulation of B and T lymphocytes, this property is associated with the ability of* M. pneumoniae* to stimulate production of cytokines and antibodies in the acute inflammatory response. The inhibitory effect of IFKOS on induction of specific MP-IgM might be related to affect the function of B lymphocytes.

Quantitative real-time PCR was used to investigate the ability of MP to infect animals and the therapeutic effect of IFKOS. The results showed that the relative expression of MP-DNA in the lung tissues of the infection group was obviously higher than in the lung tissues of the other groups. AZM treatment significantly reduced the relative expression of MP-DNA compared with the infection group, whereas IFKOS showed no obvious inhibitory effect on it. The level of MP-IgM in the infection group increased significantly compared to the normal control group. IFKOS treatment obviously decreased the level of MP-IgM in a dose-dependent manner, which indicated that elevation of MP-specific IgM was significantly depressed by IFKOS treatment in a dose-dependent manner. The results suggest that IFKOS strongly inhibits acute infection induced by MP in infant mice, the mechanism may be related to the suppression of the IgM response induced by MP rather than directly inhibiting MP replication.

Some cases of mycoplasma pneumoniae pneumonia result in fulminant respiratory failure and are fatal in children [40]. In a previous study, the actual mortality rate in fulminant MPP cases was reported to be 3–5% [41]. In our study, the mortality rate, survival rate, death protection rate, survival time, and life-prolonging rate in BALB/c mice model were calculated to estimate the protective effect of IFKOS on severe MP pneumonia. The results demonstrated that IFKOS treatment significantly decreased mortality rate and dramatically increased survival time in a dose-dependent manner, which indicated that IFKOS could protect infant mice from a lethal MP challenge.

Excessive immune responses were involved in the pathogenesis of MP pneumonia, especially in RMPP, where the pathogenesis of pulmonary injury may be due to the host's immune response rather than direct microbial damage [9, 42]. There is evidence to indicate that cytokines are closely associated with the development of mycoplasma pneumoniae infections or the severity of disease both in patients and animal models [43].

However, few studies of cytokines in Wistar rats have been reported. So in order to investigate the role of cytokines in the immunopathologic responses in MP pneumonia, we detected the changes of cytokines levels in lung tissues of an MP pneumonia rat model.

IL-2 is a proinflammatory cytokine secreted from Th1 cells and can promote a cell-mediated immune responses to kill intracellular microbial pathogens [44]. TNF-*α* is a polypeptide regulatory factor produced by Th1 cells, with proinflammatory effects on eosinophils, neutrophils, T cells, and epithelial cells, and it can cause local inflammation and even multiple organ injury [45]. IL-6 is another important proinflammatory cytokine, being a vital Th2 cytokine involved in the pathological process of pulmonary inflammation. It has been reported that IL-6 may be involved in the infection process of MP and play an important role in the pathogenesis of MP pneumonia [46].

Our study showed that IL-2 and TNF-*α* levels significantly increased while the IL-6 level obviously decreased in the lung tissues of rats infected with MP. Of greater interest in the present study is that the elevated IL-2 and TNF-*α* levels were significantly reduced by IFKOS treatment. In addition, the decreased IL-6 level was significantly enhanced by IFKOS at the doses of 10 and 5 ml/kg. The results indicated that proinflammatory cytokines IL-2, TNF-*α*, and IL-6 were simultaneously involved in MP pneumonia, in the progress of which Th1 cytokines (IL-2 and TNF-*α*) were upregulated and Th2 cytokine (IL-6) was downregulated, thereby changing the balance of Th1/Th2 toward Th1 cells. A Th1/Th2 balance function plays a significant role in anti-infectious immunity and the response of Th1/Th2 cytokines are useful biomarkers in diagnosis and treatment of MP infection in children with pulmonary involvement [47, 48]. Our studies demonstrated that the imbalance of the Th1/Th2 function after* Mycoplasma pneumoniae *infection is an important immunological mechanism of MP pneumonia. IFKOS treatment could reverse the imbalance of Th1/Th2 cytokines induced by MP infection in the infant rats. It has been reported that IL-18 and IL-1ß were associated with* Mycoplasma pneumoniae* infection. We have detected the levels of IL-18 and IL-1ß in lung tissues. The levels of IL-18 and IL-1ß were significantly increased in the infection group compared with the normal control group, but IFKOS showed no obvious inhibitory effect on the elevated levels of IL-18 and IL-1ß. So the results of IL-18 and IL-1ß were not showed in the manuscript.

In conclusion, our study shows that IFKOS has an inhibitory effect on* Mycoplasma pneumoniae* infection in infant mouse and rat models of MP pneumonia and a protective effect from lethal MP challenge in an infant murine model. These anti-MP effects might be related to suppression of the IgM response and a reversal of the imbalance of Th1/Th2 cytokines induced by MP infection. These findings provide direct evidence that IFKOS represents a potential proprietary Chinese medicine to combat pediatric MP pneumonia.

## Figures and Tables

**Table 1:** Inhibitory effect of IFKOS on PI in mice MP pneumonia model.

Group	PI	IRPI(%)
Normal control	0.68±0.12	-
Infection control	1.56±0.32^*∗∗*^	-
azithromycin	1.28±0.28	32.61
IFKOS(40ml/kg)	1.08±0.12^##^	54.93
IFKOS(20ml/kg)	1.29±0.22^#^	31.57
IFKOS(10ml/kg)	1.23±0.14^##^	37.78

Each group represented the value of PI and IRPI. PI was expressed as mean ± SEM (n = 10).  ^*∗∗*^p < 0.01 compared to normal control group;  ^##^p<0.01,  ^#^p<0.05 compared to infection control group.

**Table 2:** Effect of IFKOS on pulmonary lesion of mice or rats infected with MP.

Group	Number of animals	mice		rats
		lung	bronchus	lung
Normal control	10	0	0	0
Infection control	10	2.9^*∗∗*^	2.4^*∗∗*^	2.1^*∗∗*^
azithromycin	10	2.2	2.1	1.3^#^
IFKOS(high dose)	10	2.3	2.5	1.3^#^
IFKOS(middle dose)	10	2.4	2	1.3^#^
IFKOS(low dose)	10	2.1^#^	2.4	1.4

Each group represented the mean score of pulmonary lesion of mice or rats infected with MP. ^*∗∗*^p < 0.01 compared to normal control group, ^##^p<0.01, ^#^p<0.05 compared to infection control group.

## Data Availability

The research data used to support the findings of this study are included within the article.

## Additional Points

*Impact Statement*. In the present study, the therapeutic effect and mechanisms of IFKOS on MP pneumonia were assessed using infant mouse and rat models. qRT-PCR and ELISA assay were used to evaluate the therapeutic effect of IFKOS on the expression levels of MP-DNA, MP-IgM, IL-2, IL-6, and TNF-*α* in response to MP pneumonia. In addition, pulmonary injury in mice and rats was detected by pathological examination under microscope and the mortality rate and survival time in BALB/c mice model were observed. Our study demonstrates that IFKOS has an inhibitory effect on MP infection in infant mouse and rat models of MP pneumonia and a protective effect from lethal MP challenge in an infant murine model. These anti-MP effects might be related to suppression of the IgM response and a reversal of the imbalance of Th1/Th2 cytokines induced by MP infection. These findings provide direct evidence that IFKOS represents a potential proprietary Chinese medicine to combat pediatric MP pneumonia.
